# **α**-Ketoglutarate Accumulation Is Not Dependent on Isocitrate Dehydrogenase Activity during Tellurite Detoxification in *Escherichia coli*

**DOI:** 10.1155/2013/784190

**Published:** 2013-11-25

**Authors:** Claudia A. Reinoso, Vasu D. Appanna, Claudio C. Vásquez

**Affiliations:** ^1^Departamento de Biología, Facultad de Química y Biología, Universidad de Santiago de Chile, Santiago 9170000, Chile; ^2^Department of Chemistry and Biochemistry, Laurentian University, Sudbury, ON, Canada P3E 2C6

## Abstract

Tellurite is toxic to most microorganisms because of its ability to generate oxidative stress. However, the way in which tellurite interferes with cellular processes is not fully understood to date. In this line, it was previously shown that tellurite-exposed cells displayed reduced activity of the **α**-ketoglutarate dehydrogenase complex (**α**-KGDH), which resulted in **α**-ketoglutarate (**α**-KG) accumulation. In this work, we assessed if **α**-KG accumulation in tellurite-exposed *E. coli* could also result from increased isocitrate dehydrogenase (ICDH) and glutamate dehydrogenase (GDH) activities, both enzymes involved in **α**-KG synthesis. Unexpectedly both activities were found to decrease in the presence of the toxicant, an observation that seems to result from the decreased transcription of *icdA* and *gdhA* genes (encoding ICDH and GDH, resp.). Accordingly, isocitrate levels were found to increase in tellurite-exposed *E. coli*. In the presence of the toxicant, cells lacking *icdA* or *gdhA* exhibited decreased reactive oxygen species (ROS) levels and higher tellurite sensitivity as compared to the wild type strain. Finally, a novel branch activity of ICDH as tellurite reductase is presented.

## 1. Introduction

Tellurium is a metalloid belonging to the chalcogen group of the Periodic Table of elements and is found in elemental (Te^0^), inorganic [telluride (Te^2−^), tellurite (TeO_3_^2−^), tellurate (TeO_4_^2−^)] and organic [dimethyl telluride (CH_3_TeCH_3_), dimethyl ditelluride (CH_3_TeTeCH_3_)] forms [1].

In particular, tellurite is toxic for most living organisms because of its ability to generate oxidative stress. This was first demonstrated in *Escherichia coli* exposed to the toxicant, where increased levels of intracellular reactive oxygen species (ROS), particularly superoxide, were observed [2–6]. With the exception of glutathione [7], most intracellular tellurite targets are still poorly understood. Nevertheless, it has been established that tellurite-exposed *E. coli* results in decreased activity of certain ROS-sensitive enzymes from the Krebs cycle such as aconitase and fumarase [3, 8]. Furthermore, *Pseudomonas fluorescens* and HepG2 cells exposed to ROS-producing metals exhibit decreased *α*-ketoglutarate dehydrogenase (*α*-KGDH) and increased isocitrate dehydrogenase (ICDH) activity. These conditions result in *α*-ketoglutarate (*α*-KG) accumulation, Krebs cycle disruption, and decreased NADH and ATP levels, suggesting that *α*-KG may be involved in the cellular response to these ROS elicitors [9]. *α*-KG accumulation, as consequence of enhanced glutamate dehydrogenase (GDH) activity, was also observed in menadione-exposed *P*. *fluorescens* [10]. 

On the other hand, hydrogen peroxide oxidizes *α*-keto acids yielding CO_2_ and water [12], a reaction underlying the cytoprotective effect of *α*-keto acids such as pyruvate and *α*-KG [13]. These molecules also protect against *tert*-butyl hydroperoxide (*tert*-BuOOH) induced oxidative damage [14]. 

In addition to decreasing ROS, hydrogen peroxide-mediated *α*-KG oxidative decarboxylation helps to compensate succinate levels upon *α*-KGDH complex inhibition during oxidative stress, thus alleviating the Krebs cycle function [15]. In this context, decreased *α*-KGDH activity and consequently increased *α*-KG content were observed in tellurite-exposed *E. coli* [16]. However, *α*-KG accumulation could also result from increased ICDH and/or GDH activity. ICDH catalyzes the oxidative decarboxylation of isocitrate to *α*-KG yielding the important reducing equivalent NADPH [17]. In most bacteria, ICDH is a dimeric enzyme consisting of identical subunits with a molecular mass of ~45 kDa [18]. NADPH is also essential for regenerating reduced glutathione (GSH) by glutathione reductase [19], an important reaction protecting cells from oxidative damage. In this context, ICDH could also play an antioxidant role during oxidative stress [20]. In turn, GDH catalyzes the reversible reductive amination of *α*-KG to yield glutamate. In *E. coli*, the enzyme consists of six identical polypeptides [21].

This work was undertaken to analyze if the *α*-KG-synthesizing enzymes ICDH and GDH were responsible or not for the increased *α*-KG levels observed in tellurite-exposed *E. coli*. 

## 2. Materials and Methods

### 2.1. Bacteria, Growth Conditions, Toxicant Tolerance and Isocitrate Content Determination

Bacterial strains used in this work are listed in Table 1. Cells were routinely grown in Luria-Bertani (LB) or M9 minimal medium [22] at 37°C with shaking. Growth was started by inoculating 1 : 100 dilutions of overnight cultures into fresh medium. When required, kanamycin (100 *μ*g/mL) was amended to the medium. Growth inhibition zones (GIZ), minimal inhibitory concentrations (MIC), and intracellular isocitrate levels were determined as described previously for *α*-KG [16]. Protein concentration was determined as described by Bradford [23].

#### 2.1.1. qRT-PCR

Cells were grown in LB medium to OD_600_~0.5 and exposed to 0.5 *μ*g/mL tellurite (30 min). Total RNA was purified using the RNAsy kit (Qiagen) as recommended. Two *μ*g of purified RNA was used as template and reactions were carried out using the LightCycler RNA Amplification SYBR Green I kit (Roche Applied Science). Specific primers used are indicated in Table 1. Transcript amounts (ng) of *gdhA *and* icdA* mRNA were calculated using standard curves made with known template concentrations. *rpoD* mRNA was used as the housekeeping gene for normalization.

### 2.2. ROS Monitoring by Flow Cytometry

*E. coli* BW25113, Δ*gdhA*, and Δ*icdA* strains grown to OD_600_~0.5 were exposed to 0.05 *μ*g/mL tellurite for 30 min to determine total ROS. After washing, centrifuging, and suspending in 500 *μ*L of 25 mM phosphate buffer pH 7.0 (buffer A), cells were incubated for 30 min in the dark with 0.02 mM 2,7 dihydrodichlorofluorescein diacetate (H_2_DCFDA). Fluorescence intensity (*λ*_ex_ 428, *λ*_em_ 522) was monitored as described previously [16]. Cells exposed to 5 mM TBH (*tert*-butyl hydroperoxide) were used as positive control of oxidative stress. 

To assess superoxide, the referred strains were grown to OD_600_~0.5 and exposed to 0.05 *μ*g/mL tellurite for 30 min. After centrifuging and washing with buffer A, cells were suspended in 500 *μ*L of buffer A and incubated in the dark for 15 min with 0.05 mM dihydroethidine (DHE). Intensity was assessed using a Becton Dickinson (model FacsCanto II) apparatus equipped with an Argon laser (*λ*_ex_ 520, *λ*_em_ 610). Tellurite-exposed Δ*sodAsodB E. coli *was used as control.

### 2.3. Determination of Enzymatic Activity

GDH was assayed in cell-free extracts from tellurite-exposed *E. coli* (0.5 *μ*g/mL, 30 min) at 37°C. NADP^+^ reduction was monitored at 340 nm for 1 min. The reaction mixture (1 mL) contained 25 mM Tris-HCl buffer, pH 7.0, 0.5 mM NADP^+^ and 10 mM glutamate. Assays were started with the extract (100 *μ*g protein). Blue native polyacrylamide gels were run for in-gel visualization of enzyme activity, coupling NADH/NADPH formation to 0.3 mg/mL phenazine methosulfate and 0.5 mg/mL iodonitrotetrazolium as described [24].

ICDH was assessed at 37°C in cell-free extracts from tellurite-exposed *E. coli* (0.5 *μ*g/mL, 30 min). Blue native polyacrylamide gels were run and incubated with 25 mM Tris-HCl pH 7.4 buffer containing 5 mM MgCl_2_ for 15 min. To visualize ICDH activity, gels were immersed into a solution containing 5 mM isocitrate and 0.5 mM NADP^+^. Enzymatic activity was detected in gels by formazan precipitation from 0.4 mg/mL iodonitrotetrazolium and 0.2 mg/mL phenazine methosulfate [24].

Tellurite reductase (TR) activity associated to ICDH was determined by fractionating 100 *μ*g protein through non-denaturing polyacrylamide gel electrophoresis. After the run, gels were washed, immersed into a solution that contained 1 mM K_2_TeO_3_ and 1 mM NADH, and incubated at 37°C. 

### 2.4. Data Analysis

In general, results were expressed as the mean ± the standard deviation. Differences between experimental groups were analyzed using one-way ANOVA. *P* values < 0.05 were considered statistically significant.

## 3. Results and Discussion 

In spite of many efforts, the overall effects of tellurite in TeO_3_^2−^-exposed cells are not fully understood to date. In this context, some work has been done to unveil the toxicant's effect on several bacterial metabolic pathways [5]. Important data were obtained while studying the interaction of tellurite with the electron transport chain via the DsbB link to the quinone pool in *Rhodobacter capsulatus *[25]. Shortly after that, it was communicated that the E3 component (dihydrolipoyl dehydrogenase) of the *E. coli* pyruvate dehydrogenase complex is able to reduce tellurite to its elemental, metallic form [26]. More recently, it was shown that tellurite-exposed *E. coli* exhibits decreased activity of the key glycolytic enzymes pyruvate kinase (PK) and phosphofructokinase (PFK) [27]. However, not much is known about tellurite effects on the Krebs cycle. Some minor data regarding dissipation of the transmembrane ΔpH gradient resulting in lower ATP levels in tellurite-exposed *E. coli *[28] and inactivation of fumarase and aconitase [8] have been published. 

### 3.1. Effects of Tellurite on *E. coli* ICDH and GDH Activity and *gdhA* and *icdA* Gene Transcription

**Figure 1:**
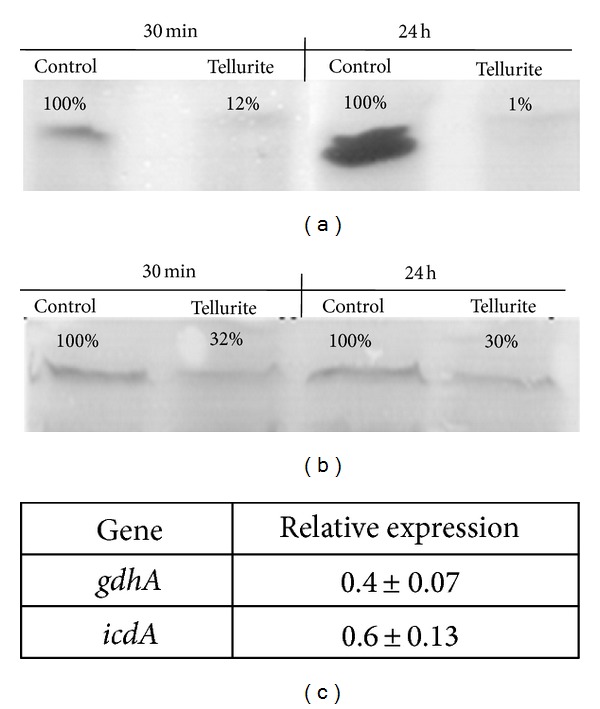
*In situ* ICDH and GDH activity and transcriptional level of *gdhA* and *icdA* genes in tellurite-exposed* E. coli*. ICDH (a) and (b) GDH activity was assayed after fractionating extracts from tellurite-treated cells by native gradient polyacrylamide gels as described in methods. Representative gels are shown. (c) Transcriptional level of *gdhA* and *icdA* genes in tellurite-exposed *E. coli*. Numbers represent the mean of 3 independent trials. The relative expression was normalized to that of the *rpoD* gene.

Previous work showed that *α*-KG accumulates in response to *E*. *coli* exposure to tellurite [16]. Since accumulation of this *α*-keto acid could also result from increased ICDH and/or GDH activities, these enzymes were analyzed in cell-free extracts from tellurite-exposed wild type *E*. *coli*. After fractionation by native PAGE, *in situ* activity assays showed a significant decrease of ICDH activity, as seen previously for *α*-KGDH [16] (Figure 1(a)). Using the same experimental approach, GDH activity was also found severely decreased (~70%) (Figure 1(b)). None of these activities were recovered after exposing *E*. *coli* to tellurite for 24 h. These results clearly show that *α*-KG accumulation does not result from an increase of these activities. Decreased ICDH activity has also been observed in cells exposed to nitric oxide [29], peroxynitrite [30], ROS [31], and lipid peroxidation products [29, 32]. *In vitro* experiments with purified ICDH also demonstrated that it loses activity if exposed to H_2_O_2_, superoxide or hydroxyl radicals, and photochemically-generated singlet oxygen [31, 33]. In this context, the decreased ICDH activity observed in tellurite-exposed *E*. *coli* could be a consequence of the superoxide anion that is generated during tellurite reduction in TeO_3_^2−^-exposed cells [3, 8]. Although it has been shown that GDH activity declines with H_2_O_2_ [34], it is too preliminary to assign this enzyme some responsibility in *α*-KG intracellular accumulation in the presence of tellurite. In this line, the increased H_2_O_2_ levels detected in TeO_3_^2−^-exposed cells could explain the observed decrease of GDH activity.

To test if the observed decrease of ICDH and GDH activities was reflected at the transcriptional level, qRT-PCR assays were carried out as described in Methods. Results showed that *icdA* and *gdhA* expression was repressed in tellurite-exposed *E*. *coli* (Figure 1(c)), suggesting that tellurite effects may occur at the protein level or as a consequence of *α*-KG accumulation. 

**Figure 2:**
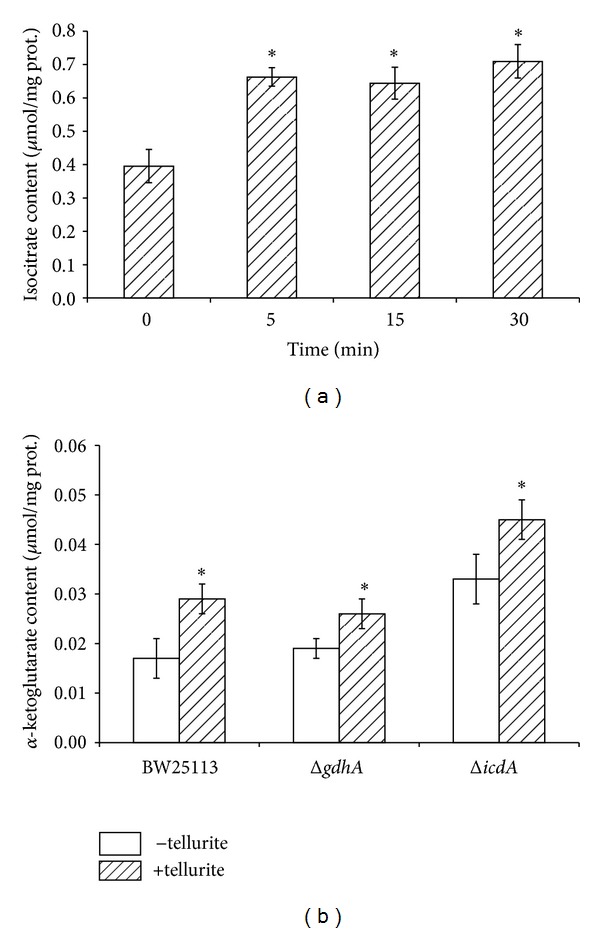
Isocitrate and *α*-KG content in tellurite-exposed *E. coli*. (a), Isocitrate content was assessed by HPLC in extracts from wild type *E. coli *previously exposed to 0.5 *μ*g/mL tellurite for 5, 15 and 30 min as described in Methods. Numbers represent the mean of 3 independent trials ± SD. *P < 0.005. (b), *α*-KG content in *E. coli* previously grown in LB medium. *α*-KG content was determined by HPLC in cell-free extracts of the indicated *E. coli* strains as described in Methods. Bars represent the standard deviation (*n* = 3). *P < 0.005.

One would expect that a decrease of ICDH activity results in isocitrate accumulation. This was precisely the case; that is, this tricarboxylic acid accumulates quickly after tellurite exposure (Figure 2(a)). Another enzyme that could help to explain isocitrate accumulation is isocitrate lyase (ICL), which catalyzes the reversible reaction isocitrate ↔ glyoxylate + succinate [35]. Conversely to the increased ICL observed in aluminium-exposed *P*. *fluorescens* [36], ICL decreased in tellurite-exposed *E*. *coli* supporting the idea that isocitrate accumulation could also result from decreased ICL activity (unpublished data). Thus, the activity of some enzymes from the Krebs cycle decreases in the presence of the toxicant, which in turn may explain the decrease of ATP levels in tellurite-exposed *E*. *coli* [28].

### 3.2. ICDH Displays Tellurite Reductase (TR) Activity

**Figure 3:**
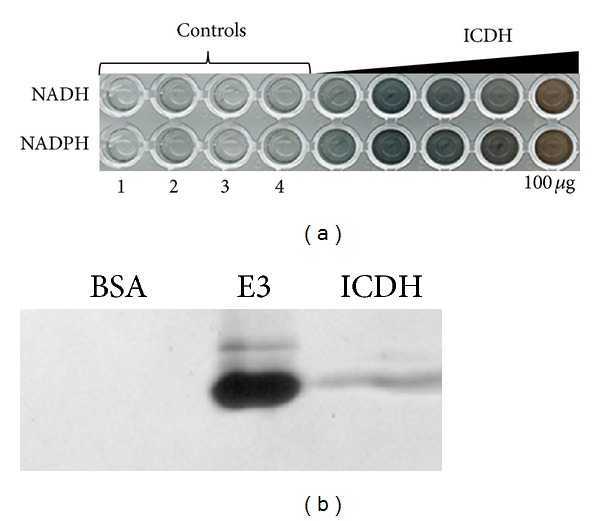
ICDH's tellurite reductase activity. (a) TR activity determined by plaque assay. Controls are (1) only buffer, (2) buffer + 1 mM *β*-mercapto ethanol (*β*-ME), (3) buffer + *β*-ME + 1 mM tellurite, and (4) buffer + *β*ME + tellurite + bovine serum albumin (BSA). Purified ICDH was added at 5, 10, 25, 50, and 100 *μ*g, as indicated in the figure. (b) TR activity was revealed after native polyacrylamide gel electrophoresis as described in methods. A representative gel is shown. BSA and purified dihydrolipoyl dehydrogenase (E3) from *Aeromonas caviae* were used as negative and positive controls, respectively.

Regarding bacterial tellurite resistance and given that some dehydrogenases such as pyruvate dehydrogenase (PDH) exhibit a branch activity related to Te^4+^→Te^0^ reduction [26] it was interesting to test if ICDH also displayed such a TR activity. ICDH's TR activity was observed either in plaque assays or after fractionation by native PAGE (Figures 3(a) and 3(b)). If the ICDH ability to reduce tellurite is related or not to the observed decrease of ICDH activity is not yet fully understood. Maybe ICDH's TR activity could be activated to eliminate tellurite, but also decreased ICDH activity-mediated isocitrate accumulation could act as a signal to decrease the efficiency of the Krebs cycle to limit NADH generation and thus ROS production in the presence of the toxicant.

### 3.3. Tellurite Tolerance and ROS Generation in Δ*gdhA* and Δ*icdA* Strains

**Figure 4:**
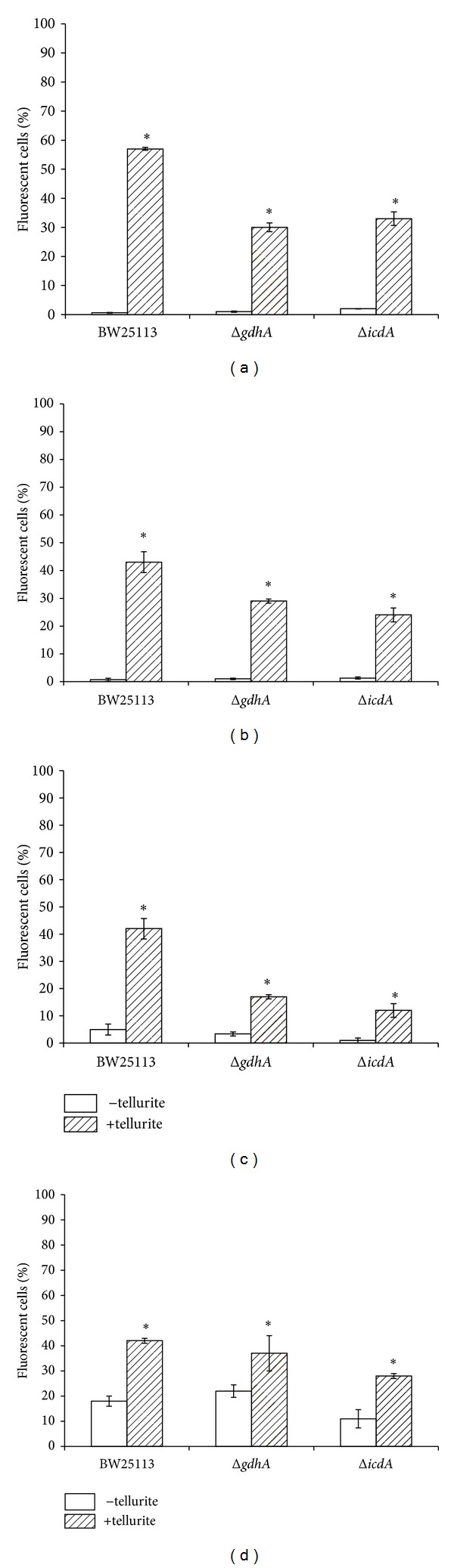
Total ROS and superoxide levels in tellurite-exposed *E. coli*. The indicated *E. coli* strains, exposed or not to tellurite, were assessed by flow cytometry to determine ROS as described in methods. ((a) and (b)): total ROS in LB-or M9-grown cells, respectively. ((c) and (d)): Superoxide levels in LB-or M9-grown cells, respectively. Numbers are the mean of 3 independent trials. *Significant regarding controls.

Since ICDH and GDH activities decrease and *α*-KG accumulates in tellurite-exposed *E*. *coli*, it was interesting to determine tellurite tolerance in strains lacking the *icdA* or *gdhA* gene. Toxicant tolerance was assessed by determining growth inhibition zones (GIZ) and minimal inhibitory concentrations (MIC). Both mutant strains showed greater sensitivity to tellurite than the isogenic, parental, wild type strain (Table 2). This higher sensitivity is most probably due to the metabolic changes occurring in the absence of the referred genes. In fact and regarding the Δ*icdA* strain, delayed growth and decreased glucose consumption along with declined generation of NADPH and ATP have been observed [37]. Since it is expected that *α*-KG accumulation protects against oxidative stress, total ROS and superoxide were assessed in tellurite-exposed *E*. *coli* strains by flow cytometry using H_2_DCFDA [38, 39] and DHE [38] probes, respectively. In general and irrespective of the culture medium, all tested strains showed increased ROS and superoxide levels in the presence of tellurite. Lower ROS and superoxide levels observed in Δ*icdA* (Figure 4 and Figures S1–S4) are most probably explained because these cells accumulate higher amounts of *α*-KG than the parental strain (Figure 2(b)).

Since *α*-KG levels can also increase by means of aspartate aminotransferase (L-aspartate + *α*-KG ↔ oxaloacetate + L-glutamate [40] and/or glutamate synthase (L-glutamine + *α*-KG + NADPH + H^+^  ↔ 2 L-glutamate + NADP^+^ [41], experiments aiming to address the participation of these enzymes in *α*-KG accumulation in tellurite-exposed cells are under way in our laboratory. 

**Scheme 1:**
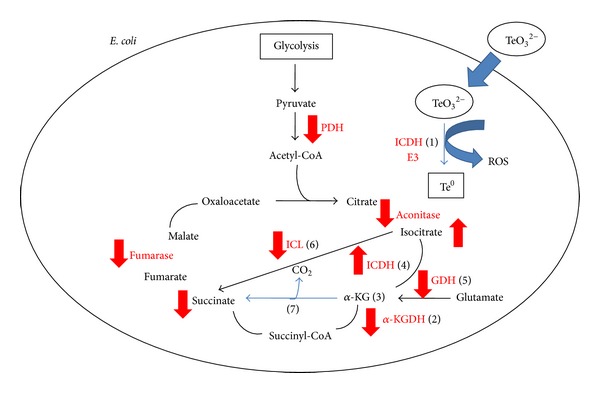
General model for the* E. coli* response to tellurite-mediated oxidative stress. See text for details.

Finally, the results from this work and from other groups regarding this issue are summarized in Scheme 1. Once tellurite enters the cell, it becomes reduced by a number of tellurite reductases (TRs) such as ICDH and E3 (from *α*-KGDH or PDH multienzyme complexes) [1], thus increasing intracellular ROS. E3's TR activity and the observed, diminished,* sucA* (encoding E1 from *α*-KGDH) transcriptions provoke decreased *α*-KGDH activity and *α*-KG accumulation [2, 3]. In addition, ICDH, GDH, ICL, PDH, fumarase, and aconitase activities are decreased when the cell faces tellurite [4–6], generating increased *α*-KG content, which could be non-enzymatically decarboxylated in the presence of hydrogen peroxide [7].

## Supplementary Material

To support the results of figure 4, supplementary figures S1 and S2 correspond to Dot Plots of total ROS detected in tellurite-exposed *E. coli* and are equivalent to those shown in figures 4A and B, respectively. In turn, supplementary figures S3 and S4 represent superoxide detection are equivalent to figures 4C and D, respectively.
Figure S1. Total ROS in *E. coli* grown in LB medium and exposed to tellurite. *E. coli* cells exposed or not to tellurite or tert-butyl hydroperoxide (TBH) were assessed for total ROS by flow cytometry using 2′,7′-dihydrodichlorofluorescein diacetate as described in Methods. X axis, fluorescence intensity, Y axis, forward scattering.
Figure S2. Total ROS in *E. coli* grown in M9 minimal medium and exposed to tellurite. See legend to figure S1 for details.
Figure S3. Superoxide levels in *E. coli* grown in LB medium and exposed to tellurite. *E. coli* cells exposed or not to tellurite were assessed for superoxide by flow cytometry using dihydroethidine as described in Methods.
Figure S4. Superoxide levels in *E. coli* grown in M9 minimal medium and exposed to tellurite. See legend to figure S3 for details.

## Figures and Tables

**Table 1:** *E.  coli* strains and primers.

Strain	Relevant genotype	Reference
BW25113	*lacIq rrnB *Δ*lacZ hsdR514 *Δ*araBAD *Δ*rhaBAD *	[11]
Δ*gdhA *	BW25113 Δ*gdhA (gdhA::kan) *	[11]
Δ*icdA *	BW25113 Δ*icdA (icdA::kan) *	[11]
Δ*sodAB *	DE(*lac*)4169 *rpsL *DE(*sodA*-*lacZ*)49 DE(*sodB*-*kan*)1-DE(2) Cam^R^ Kan^R^	[11]
*icdA*pCA24N	BW25113 carrying pCA24N	[11]

Primers	Forward (F) or reverse (R), to amplify	5′-3′ sequence

*gdhA* F	F, *gdhA *	CATATTCTCTGGAGTCATTCCTCA
*gdhA* R	R, *gdhA *	ATCATCAACCCA TACCACGC
*icdA* F	F, *icdA *	TCCGGCACAAGGCAAGAAGA
*icdA* R	R, *icdA *	CAGCCAGACGTCCTGACCAT

**Table 2:** Tellurite growth inhibition zone (GIZ) and minimal inhibitory concentration (MIC) for the indicated *E. coli* strains.

Strain	GIZ (cm^2^)		MIC (*µ*g/mL)	
	Culture medium			
	LB	M9	LB	M9
BW25113	7.12 ± 0.3	4.91 ± 0.05	0.8 ± 0.09	12 ± 0.89
Δ*icdA *	8.81 ± 0.1	7.63 ± 0.2	0.2 ± 0.03	2 ± 0.22
Δ*gdhA *	8.24 ± 0.05	6.6 ± 0.2	0.2 ± 0.03	2 ± 0.22

Numbers represent the mean of 3 independent trials ± SD.
